# How Can We Encourage Older Adults to Adopt Digital Services?

**DOI:** 10.1093/geroni/igab046.3614

**Published:** 2021-12-17

**Authors:** Yutong Xie, Jiayi Wu, W Quin Yow

**Affiliations:** 1 River Valley High School, Singapore, Singapore; 3 Singapore University of Technology & Design, Singapore, Singapore

## Abstract

Majority of older adults (OA) do not use technology frequently. For example, 84% of Singapore OA own a smartphone, however, only 22% have tried apps like online shopping. Similarly, 42% of US OA own a smartphone, yet one-third of them have never used the internet. Past research indicated poorly designed user interfaces as a barrier to technology use. Based on the Technology Acceptance Model (TAM), we hypothesize that OA-friendly user interfaces can increase perceived usefulness (PU) and perceived ease of use (PEOU) of technology, which would translate into increased digital services adoption rate. Forty healthy participants aged 65 to 86 (6 males, 34 females, mean age = 72.3) were recruited through OA activity centers in Singapore and assigned to either control or experimental groups. Both groups used an online website to perform specific shopping tasks and answered a TAM questionnaire. The control group used a website modeled after current websites while the experimental group used an OA-friendly website designed based on literature review and pre-survey, with features like OA-friendly mode, tutorial and online helpdesk. Data analysis was conducted using an independent sample t-test and bootstrapping. The experimental group showed a significantly higher proficiency in performing tasks (p=0.037), PEOU (p=0.049), and PU (p=0.044) than the control group. This suggests that user interfaces friendlier to OA increases their acceptance of technology, which could translate into a higher digital service adoption rate. Therefore, this research sets the basis for further research on digital services guidelines for older adults, including for telehealth and others.

